# Retraction: NF-κB inhibitors attenuate MCAO induced neurodegeneration and oxidative stress—A reprofiling approach

**DOI:** 10.3389/fnmol.2023.1275831

**Published:** 2023-08-21

**Authors:** Frontiers Editorial Office

The journal retracts the 24 March 2020 article cited above.

Following publication, concerns were raised regarding the integrity of the images in the published figures, with areas of image duplication and manipulation in Figures 5, 6. The authors failed to provide a satisfactory explanation during the investigation, which was conducted in accordance with Frontiers' policies. As a result, the data and conclusions of the article have been deemed unreliable and the article has been retracted.

This retraction was approved by the Chief Editors of Frontiers in Molecular Neuroscience and the Chief Executive Editor of Frontiers. The authors did not agree to this retraction.

